# Plant essential oils combined with organic acids restored lipopolysaccharide-induced leaky intestine via gut microbial modulation in weaned piglets

**DOI:** 10.1016/j.aninu.2024.04.020

**Published:** 2024-06-28

**Authors:** Xiaoyu Zheng, Yibo Wang, Xuemei Zhou, Tanyi Deng, Yueqi Zhao, Zhichao Fu, Yulong Wei, Wen Ma, Shihai Zhang, Wutai Guan, Fang Chen

**Affiliations:** a State Key Laboratory of Swine and Poultry Breeding Industry, College of Animal Science, South China Agricultural University, Guangzhou 510642, China; b Guangdong Laboratory for Lingnan Modern Agriculture, South China Agricultural University, Guangzhou 510642, China; c College of Animal Science and National Engineering Research Center for Breeding Swine Industry, South China Agricultural University, Guangzhou 510642, China

**Keywords:** Weaned piglet, Essential oil, Organic acid, Gut microbiota, Lipopolysaccharide

## Abstract

Intestine derived lipopolysaccharide (LPS) is closely related to systemic inflammation and disorders, yet little is known about its roles in the weanling stress of piglets and its potential as a nutritional intervention target. This study aimed to investigate the potential of essential oils (EO) and organic acids (OA) in mitigating weaning stress in piglets by modulating the circulation of intestine derived LPS. Seventy-two weaned piglets at 21 d old with body weight of 8.12 ± 0.168 kg were randomly divided into a control group (CON) and an experimental group, each consisting of six pens with six piglets per pen, and were fed either a basal diet or a basal diet supplemented with 3 kg/t OA + 500 g/t EO (EO + OA). On the 14th day of the feeding trial, 12 weaned piglets were randomly selected from the CON group, and 6 piglets were selected from the experimental group. Based on diet composition and stress treatment, these 18 piglets were divided into the following three groups: 1) CON group. Piglets were fed a basal diet and received an intraperitoneal injection of saline as a control. 2) LPS group. Piglets were fed a basal diet and received an intraperitoneal injection of LPS (100 μg/kg body weight) to induce stress. 3) EO + OA + LPS group. Piglets were fed a basal diet supplemented with EO and OA and received an intraperitoneal injection of LPS (100 μg/kg body weight) to induce stress. The results showed that EO + OA significantly ameliorated the oxidative imbalance and inflammation disorder induced by LPS in piglets' serum and intestine by inhibiting the activation of the Toll-like receptor 4 (TLR4)/nuclear factor-kappa B (NF-κB)/mitogen-activated protein kinase (MAPK) signaling pathway. Furthermore, compared to the LPS group, supplementation with EO + OA restored LPS-induced reductions in Bcl-2 protein expression in the piglets' intestines (*P* < 0.05) and mitigated morphological damage; it also enhanced both the protein expression and relative gene expression of the tight junction proteins occludin and claudin-1 (*P* < 0.05), and reduced the plasma diamine oxidase activity (DAO) and LPS content (*P* < 0.05). Compared to the CON group, supplementation with EO + OA altered the composition of the intestinal microbiota, increasing beneficial bacteria relative abundance (*Faecalibacterium*) (*P* < 0.05) and decreasing harmful bacteria relative abundance [*Rikenellaceae_RC9_gut_group* (*P* < 0.01), *Negativibacillus* (*P* < 0.05)]. Further analysis revealed that plasma LPS content in piglets was negatively correlated with the relative abundance of *Faecalibacterium* (r = −0.662, *P* = 0.021), *Akkermansia* (r = −0.492, *P* = 0.031), and average daily gain (ADG) (r = −0.912, *P* = 0.041). Plasma LPS content was also positively correlated with the plasma inflammatory factors interleukin (IL)-1β (r = 0.591, *P* = 0.021), IL-6 (r = 0.623, *P* = 0.021), IL-12 (r = 561, *P* = 0.031) contents, and the relative abundance of *Negativibacillus* (r = 0.712, *P* = 0.041). In summary, the addition of EO + OA prevents the leakage of intestine derived LPS into the circulation by improving intestinal integrity and microbiota composition, thereby enhancing antioxidant and anti-inflammatory abilities and growth performance of weaned piglets.

## 1 Introduction

Early weaning, a common practice in the pig industry to improve economic efficiency, exposes piglets to significant stressors such as separation from sows, dietary changes, and environmental adjustments (Moeser et al., 2017; Smith et al., 2010; Xiang et al., 2020). The immature development and functionality of their intestinal tract during this period exacerbate intestinal disturbances, heighten susceptibility to pathogens, and inflammatory responses, ultimately leading to stunted growth and mortality (Beaumont et al., 2021; Smith et al., 2010). The intestinal barrier safeguards gut health by partitioning external substances from the internal environment, and its disruption results in increased intestinal permeability, allowing endotoxins and other bacterial products to leak into the bloodstream (Allam-Ndoul et al., 2020; Chopyk and Grakoui, 2020). Recent research has revealed that dysbiosis in the gut microbiota leads to the release of lipopolysaccharides (LPS), a toxic bacterial metabolite, triggering systemic inflammation and contributing to stunted growth and health issues in various animals (Agus et al., 2021; Hills et al., 2019). These research findings prompt us to question whether nutritional interventions can reduce elevated circulating LPS contents induced by gut microbiota dysbiosis and compromised intestinal barrier during weaning, thereby mitigating stunted growth.

Plant essential oils (EO) and organic acids (OA) are commonly used feed additives in the diet of weaned piglets, known for their ability to increase antioxidant levels and mitigate stress-related damage (Frame et al., 2020; Lauridsen, 2019; Vardar-Unlu et al., 2003). Essential oils, derived from plants, are frequently incorporated into human and animal diets to improve intestinal and overall health by regulating oxidative balance, due to their antimicrobial, antioxidant, and anti-inflammatory properties (Vardar-Unlu et al., 2003). Organic acids are another type of functional additive with weak acidic properties and have been shown to improve growth performance and intestinal health and in weaned piglets through antimicrobial action (Li et al., 2018). It's widely acknowledged that combinations of EO and OA enhance intestinal microecology by increasing the abundance of beneficial microbes and reducing harmful flora (Gabler et al., 2018; Xiao et al., 2021). Other studies revealed that supplementation of EO and OA strengthens the intestinal barrier by upregulating the expression of integrity-related proteins such as zonula occludens-1(*ZO*-1) and occludin (Ma et al., 2022). Therefore, we speculate that the benefits of EO and OA supplementation on growth performance may be attributed to reduced circulating LPS contents, achieved by modulating gut microbiota composition and enhancing intestinal integrity, thereby reducing the translation of intestinal-derived LPS into the bloodstream.

In this study, we initially evaluated the impact of EO and OA on the growth performance of weaning piglets. Subsequently, we employed an LPS challenge model to mimic the acute inflammatory response and the elevated LPS circulation levels induced by weanling stress, aiming to further explore the mitigating mechanisms underlying their benefits. We conducted a comprehensive analysis, encompassing antioxidant and inflammatory states, intestinal barrier integrity, and microbiota composition, to thoroughly investigate the impact of the mixture on intestinal function. Then, correlation analyses were performed on plasma LPS content with altered intestinal microbiota, intestinal permeability, as well as antioxidant and inflammatory markers, aiming to explore the potential causative relationship between intestinal-derived LPS and growth performance.

## 2 Materials and methods

### 2.1 Animal ethics statement

All animal experiments were conducted following the guidelines of the Animal Ethics Committee of South China Agricultural University and were approved by the Animal Ethics Committee of South China Agricultural University (approval no: 20210107-1, Guangzhou, China).

### 2.2 Materials and diet ingredients

The EO and OA used in this experiment were provided by DSM (Shanghai, China). The main components of the OA mixture are 11% formic acid, 13% ammonium formate, 5% acetic acid, and 8% propionic acid, with the remainder being a carrier (silicon dioxide). The effective components of the EO mixture are 2% thymol, 1% eugenol, 1% carvacrol, 1% cinnamaldehyde, and 95% carrier (silicon dioxide). The formulation of the basal diet met or exceeded the nutrient requirements recommended by the NRC (2012). The composition and nutrient levels of the basal diet is presented in Table 1. The energy content of feed and feces was determined using an oxygen bomb calorimeter (Model 6400, Parr Instruments, USA). All nutrient measurements are in accordance with China national standards. Calcium contents were assessed in accordance with the GB/T 6436-2018, employing the ethylenediaminetetraacetic acid (EDTA) complexometric titration method. The total phosphorus content in the diet was determined according to the GB/T 6437-2018 method, using the vanadium-molybdenum yellow colorimetric method and measured with a spectrophotometer. Determination of crude protein was conducted following the Kjeldahl method outlined in GB/T 6432-2018, utilizing the fully automated Kjeldahl nitrogen analyzer (Kjeltec 8400, FOSS, DK). The contents of lysine, methionine, and threonine were determined by the automatic amino acid analyzer (L-8900, HITACHI, Japan) as per the GB/T 18246-2019, while tryptophan content was assessed in line with the GB/T 15400-2019.

**Table 1:** Composition and nutrient levels of the basal diet (as-fed basis, %).

Item	Content
Corn	58.60
Soybean meal	14.00
Whey powder	7.00
Fish meal	3.00
Soy protein concentrate	6.00
Extruded full-fat soybean	4.00
Glucose	2.00
Soybean oil	2.00
Dicalcium phosphate	1.00
Limestone	0.80
NaCl	0.20
L-lysine-HCl	0.55
DL-methionine	0.10
L-threonine	0.20
L-tryptophan	0.05
Premix^1^	0.50
Total	100.00
Nutrient levels^2^	
Gross energy, MJ/kg	16.21
Crude protein	19.06
Calcium	0.80
Total phosphate	0.62
Lysine	1.49
Methionine	0.42
Threonine	0.94
Tryptophan	0.25

1 Premix provided the following per kg of the basal diet: vitamin A 12,000 IU, vitamin D_3_ 2500 IU, vitamin E 30 IU, vitamin K_3_ 3.0 mg, vitamin B_12_ 0.012 mg, riboflavin 4.0 mg, pantothenic acid 15.0 mg, niacin 40.0 mg, choline chloride 400.0 mg, folic acid 0.7 mg, vitamin B_1_ 1.5 mg, vitamin B_6_ 3 mg, biotin 0.1 mg, Mn 40.0 mg, Fe 90.0 mg, Zn 100.0 mg, Cu 8.8 mg, I 0.35 mg, Se 0.3 mg.

2 Nutrient levels are measured values.

### 2.3 Experimental design

A total of 72 healthy 21-day-old weaned piglets (Duroc × Landrace × Yorkshire) were randomly assigned to two groups, each comprising 6 pens. The initial average body weight of weaned piglets was 8.12 ± 0.168 kg. In each pen, there were 6 piglets, comprised of 3 weaned males and 3 weaned females, selected based on body weight and gender. The feeding experiment spanned 14 d, during which the piglets had free access to feed and water. Environmental conditions were maintained at a constant temperature of 24-28 °C, with relative humidity controlled within 60%－70%. The dietary treatments were as follows: 1) corn-soybean meal-based basal diet (CON); 2) corn-soybean meal-based basal diet+3 kg/t OA + 500 g/t EO (EO + OA). In the feeding experiment, piglets were weighed, and their daily feed consumption was recorded. Calculations were performed as follows:

Average daily gain (ADG) = (total body weight at the end of the experiment - total body weight at the beginning of the experiment)/(number of piglets × number of experimental day);

Average daily feed intake (ADFI) = (daily feed amount per pen - daily remaining feed per pen)/(number of pigs per pen × number of experimental day);

Diarrhoea rate = 100 × number of diarrhea piglets/(number of tested piglets × trial days);

Feed/gain (F/G) = ADFI/ADG;

Heart index = heart weight (g)/body weight at slaughter (kg);

Liver index = liver weight (g)/body weight at slaughter (kg);

Spleen index = spleen weight (g)/body weight at slaughter (kg);

Kidney index = kidney weight (g)/body weight at slaughter (kg).

### 2.4 LPS challenge experiment

**Fig. 1:**
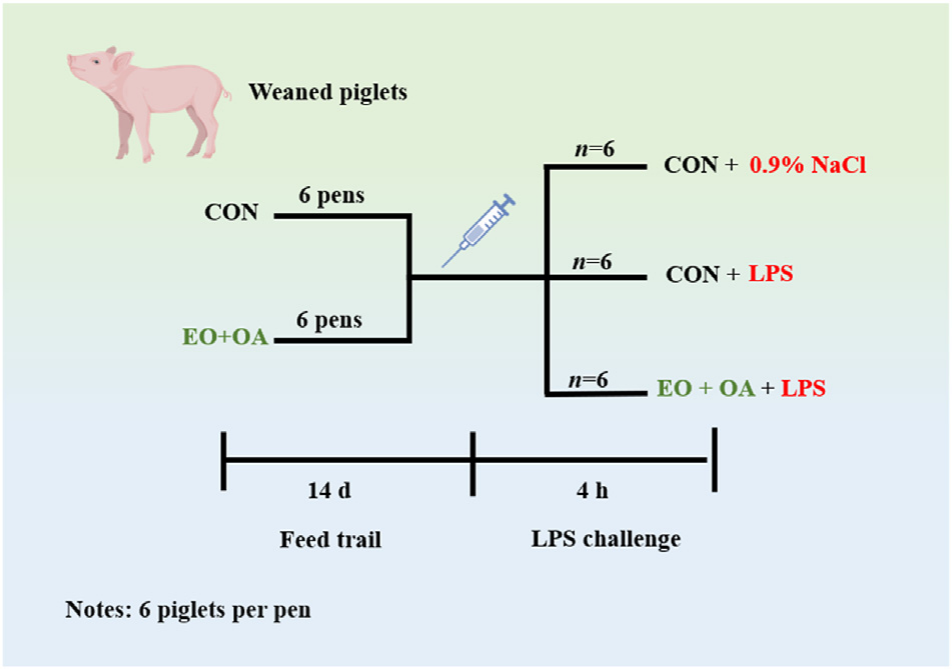
Design of experiments. CON = piglets fed the basal diet with intraperitoneal saline injection; LPS = piglets fed the basal diet and injected intraperitoneally with lipopolysaccharide (LPS) to induce stress; EO + OA + LPS = piglets fed the basal diet supplemented with essential oils (EO) and organic acids (OA) and injected intraperitoneally with LPS to induce stress.

After the feeding experiment, 18 piglets were selected for the immune stress experiment. They were divided into three groups based on their dietary composition and injection treatment, with each group comprising 6 replicates (Fig. 1). These groups include: 1) the control group (CON), in which piglets received the basal diet and underwent intraperitoneal injection of saline for challenge (injection with an equal volume of saline as the LPS challenge group). In LPS challenge experiments, injecting saline serves as a control group essential for establishing baseline comparisons. 2) The LPS group, in which piglets received the basal diet but underwent intraperitoneal injection of LPS (*Escherichia coli* 055: B5, Sigma, USA) at a concentration of 100 μg/kg. The LPS group aims to observe stress damage caused by LPS challenge in piglets without EO and OA. 3) The EO + OA + LPS group, in which piglets received an EO + OA supplemented diet along with an intraperitoneal injection of LPS at a concentration of 100 μg/kg. The objective of this group is to assess the potential impact of supplementing EO + OA on piglet immune response under LPS-induced immune stress conditions and compare it with the CON group, aiming to determine whether EO + OA supplementation can alleviate stress to a level comparable to that of the CON group.

### 2.5 Sample collection

At 14 d of the experiment, fresh feces were collected from all piglets (the aim of collecting fresh feces from all piglets for testing before the LPS challenge was to demonstrate the modulatory effect of EO + OA on gut microbiota and emphasize the protective effect of pre-supplementation with EO + OA on the gut). Following a continuous 4 h challenge with LPS or saline, samples were obtained from the randomly selected 18 weaned piglets. Blood samples were drawn from the anterior vena cava using a syringe and injected into 5 mL vacuum blood collection tubes. After standing for 1.5 h, the blood samples were centrifuged at 3500 × *g* for 15 min. The resulting supernatant was collected and stored in liquid nitrogen. Subsequently, euthanasia was performed on all piglets via injection of sodium pentobarbital (50 mg/kg body weight). The weights of the heart, liver, spleen and kidneys of each piglet were recorded. Samples of the duodenum, jejunum, and ileum were collected and preserved in 4% paraformaldehyde for morphological observations. The chyme in the jejunum tissue was washed with cold saline, and the samples were then frozen in liquid nitrogen for subsequent molecular experiments.

### 2.6 Intestinal morphology

The jejunal tissue was fixed in a 4% paraformaldehyde solution for 48 h. Subsequently, it underwent stepwise dehydration with ethanol, followed by transparency with xylene, and finally embedded in paraffin. Following that, 5 μm sections were cut at 4 °C and stained with hematoxylin and eosin. The stained sections were dehydrated again in a stepwise manner with ethanol, followed by transparency with xylene, and were subsequently coated with a sealing agent before being covered with coverslips. Each section was observed under a light microscope to measure villus height (VH) and crypt depth (CD) (NIS-Elements Viewer, Tokyo, Japan).

### 2.7 Real-time quantitative PCR

A total of 30 mg of jejunal tissue was placed into a 2.5 mL enzyme-free grinding tube, followed by the addition of 500 μL of lysis buffer, before being incubated at room temperature for 5 min for lysis. Following the lysis step, the RNA was processed through the column, underwent washing procedures, and was finally eluted following the provided instructions, resulting in the extraction of RNA (EZB-RN001-plus, EZBioscience, Shanghai, China). The concentration and purity of the RNA was determined using Nanodrop-2000. Following the instructions of the Reverse Transcription Kit with gDNA Eraser (A0010CGQ, EZBioscience, Shanghai, China) gDNA was removed and reverse-transcribed, and the resulting cDNA was stored in a refrigerator at −20 °C for later use. Real-time quantitative PCR was performed on the instrument (A28567, Thermo Fisher Scientific，China) with a total reaction volume of 20 μL. The PCR cycling conditions were as follows: an initial denaturation cycle at 95 °C for 2 min, followed by 40 amplification cycles at 95 °C for 15 s and 60 °C for 30 s. Relative target gene expression levels were determined based on a quantitative method (2^−ΔΔCt^ method) using β-actin as an internal reference, as previously described (Livak and Schmittgen, 2001). The primers used are listed in Table 2.

**Table 2:** Primer sequences used in real-time quantitative PCR.

Genes	Accession No.	Position	Primer (5′ - 3′)
*IL-1β*	NM_214055.1	Forward Reverse	TCTGCCCTGTACCCCAACTG CCAGGAAGACGGGCTTTTG
*IL-6*	AF518322.1	Forward Reverse	TGGCTACTGCCTTCCCTACC AGAGCCTGCATCAGCTCAGT
*IL-12*	NC_010458.4	Forward Reverse	CAACCCTGTGCCTTAGCAGT AGAGCCTGCATCAGCTCAGT
*TNF-α*	NM_214022.1	Forward	CCACCAACGTTTTCCTCACT
		Reverse	TAGTCGGGCAGGTTGATCTC
*TGF-β*	NM_001087861.1	Forward	ATTCCTGGCGTTACCTTGG
		Reverse	AGCCCTGTATTCCGTCTCTCCT
*TLR4*	NM_001113039.1	Forward	CAACCCTGTGCCTTAGCAGT
		Reverse	AGAGCCTGCATCAGCTCAGT
*MyD88*	NM_001099923	Forward	CGCATGGTGGTGGTTGTT
		Reverse	GCCTTCTTCATCGCCTTGTATTT
*IKKα*	NM_001114279.1	Forward	TCTTGATCCTCGGAAACCAG
		Reverse	TGCTTCGGCCCATACTTTAC
*IKKβ*	NM_001099935.1	Forward	CCTCACCTTGCTGAGTGACA
		Reverse	TCCCCACAAAGGAGGTACAG
*IκB*	EU399817.1	Forward	CTGCTCGGCAATAACACTGA
		Reverse	GAGAGGAGACCGTTGGTGAG
*ERK*	NM_021088019.1	Forward	CAGTCTCTGCCCTCCAAGAC
		Reverse	GGGTAGATCATCCAGCTCCA
*JNK*	NM _001929166.6	Forward	TGGATGAAAGGGAACACACA
		Reverse	ATGATGACGATGGATGCTGA
*P38*	NM_021091323.1	Forward	AAGACGGGGTCCTCATCTCC
		Reverse	TCTCATCGTAGGGCTCTGCT
*NFKB*	NM_001114281.1	Forward	GGGGCGATGAGATCTTCCTG
		Reverse	CACGTCGGCTTGTGAAAAGG
β-Actin	XM_021086047.1	Forward	GATCTGGCACCACACCTTCTACAAC
		Reverse	TCATCTTCTCACGGTTGGCTTTGG

*IL-1β* = interleukin-1 beta; *IL-6* = interleukin-6; *IL-12* = interleukin-12; *TNF-α* = tumor necrosis factor-alpha; *TGF-β* = transforming growth factor-beta; *TLR-4* = Toll-like receptor 4; *MyD88* = myeloid differentiation primary response gene 88; *IKKα* = IκB kinase α; *IKKβ* = inhibitor of kappa B kinase; *IκB* = inhibitor of NF-κB; *ERK* = extracellular regulated protein kinases; *JNK* = c-Jun N-terminal kinase; *P38* = p38 mitogen-activated protein kinase; *NF-κB* = nuclear factor-kappa B.

### 2.8 Western blot

A total of 80 mg of jejunal tissue from piglets was homogenized in 1.6 mL of RIPA lysis solution (P10013B, Beyotime, Shanghai, China) containing 1% protease inhibitor phenylmethylsulfonyl fluoride (PMSF) (ST507, Beyotime, Shanghai, China). The supernatant was centrifuged at 12,000 × *g* for 5 min. The protein concentration in the supernatant of jejunum homogenate was determined using a BCA protein assay kit (P0010, Beyotime, Shanghai, China). Then, equal quantities of protein (30 μg) were separated by 10% sodium dodecyl sulfate polyacrylamide gel electrophoresis (SDS-PAGE) gels (PG112, Epizyme, Shanghai, China), followed by electro transfer to a polyvinylidene difluoride (PVDF) membrane after 1.5 h at 110 V. Subsequently, the membrane was blocked with 5% skimmed milk at room temperature for 2 h. After four washes, it was then incubated overnight with the primary antibody at 4 °C. This study employed the following primary antibodies: ZO-1(21773–1-AP, Proteintech, Wuhan, China), claudin-1 (ab129119, Abcam, Cambridge, UK), occludin (27260–1-AP, Proteintech, China), phosphorylated c-Jun N-terminal kinase (P-JNK) (4688S, Cell Signaling Technology, Boston, MA, USA), JNK (9252S, Cell Signaling Technology, Boston, MA, USA), phosphorylated extracellular signal-regulated kinase (P-ERK) (9101S, Cell Signaling Technology, Boston, MA, USA), extracellular signal-regulated kinase (ERK) (1:3000, 9102S, Cell Signaling Technology, Boston, MA, USA), phosphorylated P38 (P-P38) (4511S, Cell Signaling Technology, Boston, MA, USA), P38 (8690S, Cell Signaling Technology, Boston, MA, USA), phosphorylated NF-κB (P-NF-κB) (3033S, Cell Signaling Technology, Boston, MA, USA), NF-κB (10745–1-AP, Proteintech, Wuhan, China) and β-actin (bs-0061R, Bioss, Beijing, China). After four washes with tris buffered saline with Tween-20 (TBST), the membrane was then incubated with the secondary antibody (511203, ZenBio, Chengdu, China) at room temperature for 1.5 h. Subsequently, the target bands were visualized using an Image Quant LAS 4000 mini system and an enhanced chemiluminescence kit (P1020, Applygen, Beijing, China). Bands were determined as gray values and normalized for relative protein expression levels with β-actin as internal reference using Image J software.

### 2.9 Antioxidative status

Approximately 80 mg of jejunal tissue was ground in a homogenizing grinder (Jingxin, Shanghai, China) with a 4-fold volume of ice-cold phosphate buffered saline (PBS) buffer. After centrifugation at 12,000 × *g* for 15 min, the tissue supernatant was collected and then stored at −20 °C for further analysis. The antioxidant capacity levels of plasma and jejunum in weaned piglets were assessed using superoxide dismutase (SOD) (A001-3, NJC, Nanjing, China), malondialdehyde (MDA) (A003-1, NJC, Nanjing, China), total antioxidant capacity (T-AOC) (A015-3, NJC, Nanjing, China), reduced glutathione (GSH) (A006-2, NJC, Nanjing, China), and peroxisomal glutathione (GSH-Px) (A005-1, NJC, Nanjing, China). All antioxidant index tests in plasma and jejunal samples were conducted following the manufacturer's instructions.

### 2.10 Enzyme-linked immunosorbent assays (ELISA)

The plasma samples from weaned piglets were centrifuged at 3000 × *g* for 10 min, followed by collecting the supernatant for ELISA detection. The supernatant was then incubated with the appropriate antibody for 1 h at 37 °C, followed by incubation with the provided color developer for 30 min and washing five times. Finally, the reaction was terminated by the addition of a termination solution. The concentration level of the target assay in the plasma of weaned piglets was calculated from the standard curve by measuring the optical density (OD) value at 450 nm using an enzyme marker. Plasma contents of tumor necrosis factor-alpha (TNF-α), interleukin-1 beta (IL-1β), interleukin-6 (IL-6), interleukin-12 (IL-12), LPS, and D-lactic acid (D-LA) and diamine oxidase (DAO) activity, were calculated from a standard curve according to the manufacturer's instructions (MlBio, Shanghai, China).

### 2.11 16S rRNA sequencing

Fresh fecal samples from piglets were collected and stored at −80 °C. Bacterial DNA was then isolated from the feces using the MagPure Soil DNA LQ Kit (Magen, Guangdong, China). After extracting genomic DNA from piglet fecal samples, primers 341F (5′-CCTAYGGGGRBGCASCAG-3′) and 806R (5′-GGACTACNNGGGGTATCTAAT-3′) were employed to amplify the V3 to V4 variable region of the 16S rRNA gene. DNA concentration and purity of the samples were assessed through 1% agarose gel electrophoresis. Subsequently, the qualified DNA samples underwent sequencing using an Illumina NovaSeq6000, generating paired-end reads with a cycle length of 250 bp for each end. Finally, sequencing was conducted on the Illumina Nova Seq platform, producing 250 bp paired-end reads. Subsequently, FLASH software was utilized to splice the paired-end reads, obtaining the original tag. The ASV and their feature lists were derived through filtering and noise reduction using QIME software (version 1.9.1). Species annotation of the ASV was executed to acquire species information for each ASV. The 16S rRNA gene amplicon sequencing and analysis were carried out by OE Biotechnology (Shanghai, China).

### 2.12 Statistical analysis

In this study, Student's *t*-test (SPSS 22.0) was employed to assess significant differences in growth performance between the CON and EO + OA groups, with a significance threshold set at *P* < 0.05. One-way ANOVA was employed for analyses involving more than two groups, and Tukey's multiple comparisons were conducted with a significance threshold set at *P* < 0.05. Data were expressed as the mean ± SEM. *P* < 0.01 was considered highly significant. 0.05 < *P* < 0.10 was considered a trend. Alpha indexes (ACE, Chao 1, Simpson, goods_coverage, and observed_species indexes) comparison between groups was calculated by Welch's *t*-test and Wilcoxon rank test in R project Vegan package (version 2.5.3). β-diversity was analyzed using principal coordinate analysis (PCOA) based on the Bray–Curtis distance. Species comparison between groups was calculated by Welch's *t*-test and Wilcoxon rank test in R project Vegan package (version 2.5.3). Pearson's calculations were used to construct heatmap models for correlation analyses in R (Version 3.2.4). *P* < 0.05 was deemed statistically significant, while *P* < 0.01 was considered highly significant. 0.05 < *P* < 0.10 was considered a trend. Significance is indicated as ∗*P* < 0.05 and ∗∗*P* < 0.01, denoting statistical significance levels.

## 3 Results

### 3.1 Growth performance and organ indices

The growth performance and organ indices of piglets are shown in Table 3, Table 4, Table 5. On the initial day of the study, we conducted weight measurements for all experimental piglets. Throughout the experiment, we monitored their daily feed intake. Finally, after 14 d, we performed another round of piglet weighing. The results indicate that the EO + OA group shows a trend of increased ADG compared to the CON group (*P* = 0.085), while there is a trend of decreased F/G (*P* = 0.072) (Table 3). We found that feeding piglets with EO + OA for 14 days reduced the diarrhea rate, but the difference was not significant (*P* = 0.081). Additionally, feeding EO + OA from d 15 to d 28 significantly reduced the diarrhea rate in piglets (*P* = 0.049) compare to the CON group (Table 4). There was no significant difference in ADFI between the two groups from d 1 to d 28 (*P* = 0.062). Additionally, we assessed the weights of different internal organs in the piglets at the conclusion of the experiment. Compared to the CON group, the spleen index of the LPS group increased, but the difference was not significant (*P* = 0.107) (Table 5).

**Table 3:** Effects of EO + OA on the growth performance of weaned piglets.

Item	CON	EO + OA	SEM	*P-*value
Initial weight, kg	8.35	8.08	0.168	0.167
Final weight, kg	12.46	12.99	0.298	0.122
ADG, kg	0.29	0.35	0.026	0.085
ADFI, kg	0.52	0.54	0.006	0.482
F/G	1.87	1.56	0.138	0.072
Diarrhoea rate, %	12.86	10.11	0.164	0.081

ADG = average daily gain; ADFI = average daily feed intake; F/G = feed/gain; CON = piglets fed the basic diet; EO + OA = piglets fed the basal diet supplemented with essential oils (EO) and organic acids (OA).

Means with no letter superscripts indicate no significant differences (*P*＞0.05).

**Table 4:** Effects of EO + OA on growth performance of each stage.

Item			Con	EO + OA	SEM	*P*-value
Body weight, kg						
d 1			6.54	6.48	0.251	0.971
d 14			12.51	13.19	0.492	0.083
d 28			20.25	21.70	0.532	0.072
Days 1–14						
ADG, g			449	478	16.0	0.092
ADFI, g			608	639	23.3	0.086
F/G			1.59	1.42	0.032	0.074
Diarrhea rate, %			12.19	10.11	0.672	0.076
Days 15– 28						
ADG, g			562	599	19.2	0.051
ADFI, g			886^b^	987^a^	37.3	0.043
F/G			1.62	1.48	0.053	0.062
Diarrhea rate, %			7.42^a^	6.90^b^	0.394	0.049
Days 1– 28						
ADG, g			492^b^	545^a^	14.2	0.048
ADFI, g			758	828	20.2	0.062
F/G			1.60	1.45	0.034	0.077
Diarrhea rate, %			8.99	6.69	0.523	0.068

ADG = average daily gain; ADFI = average daily feed intake; F/G = feed/gain; CON = piglets fed the basic diet; EO + OA = piglets fed the basal diet supplemented with essential oils (EO) and organic acids (OA).

^a, b^ Means with different letter superscripts indicate significant differences (*P*＜0.05), while with no letter or the same letter superscripts indicate no significant differences (*P*＞0.05).

**Table 5:** Effects of EO + OA on organ indices of weaned piglets.

Item	CON	LPS	EO + OA + LPS	SEM	*P-*value
Heart, g/kg	5.72	5.61	5.15	0.218	0.562
Liver, g/kg	29.76	32.71	36.56	1.012	0.163
Spleen, g/kg	2.32	2.96	2.59	0.142	0.107
Kidney, g/kg	5.42	6.05	6.24	0.212	0.408

CON = piglets fed the basal diet with intraperitoneal saline injection; LPS = piglets fed the basal diet and injected intraperitoneally with lipopolysaccharide (LPS) to induce stress; EO + OA + LPS = piglets fed the basal diet supplemented with essential oils (EO) and organic acids (OA) diet and injected intraperitoneally with LPS to induce stress.

Means with no letter superscripts indicate no significant differences (*P*＞0.05).

### 3.2 Plasma redox and inflammation

To assess the effects of EO + OA on the antioxidant capacity and inflammatory levels in the plasma of weaned piglets, we measured the levels of oxidative stress and inflammatory markers in plasma (Table 6). Compared to the CON group, the LPS group showed a significant decrease in SOD activity (*P* = 0.014) and a significant increase in MDA content (*P* = 0.012). However, compared to the LPS group, the EO + OA + LPS group exhibited a significant increase in SOD activity and T-AOC, along with a significant decrease in MDA content (*P* < 0.05). There were no significant differences in GSH content and GSH-Px activity between the groups (*P* > 0.05). In addition, compared to the CON group, weaned piglets exposed to LPS exhibited significantly increased contents of the pro-inflammatory factors TNF-α, IL-1β, IL-6, and IL-12 (*P* < 0.05). However, supplementation of EO + OA in the diet mitigated the elevation of TNF-α, IL-1β, and IL-6 contents induced by the LPS challenge (*P* < 0.05). There was significant effect on IL-12 content (*P* = 0.014). In summary, supplementing EO + OA in the diet of weaned piglets enhanced antioxidant and anti-inflammatory capabilities, strengthening their resistance to LPS-induced stress injury.

**Table 6:** Effects of EO + OA on plasma antioxidants and inflammatory factors in weaned piglets.

Item	CON	LPS	EO + OA + LPS	SEM	*P-*value
TNF-α, pg/mL	18.28^b^	29.70^a^	7.33^b^	3.832	0.044
IL-1β, pg/mL	621.04^b^	1083.15^a^	641.87^b^	104.653	0.035
IL-6, pg/mL	32.28^b^	143.69^a^	14.61^b^	17.298	0.026
IL-12, pg/mL	21.77^c^	48.55^a^	37.77^ab^	4.146	0.014
MDA, nmol/mL	3.01^b^	6.43^a^	4.61^b^	0.486	0.012
T-AOC, mmol/L	0.14^ab^	0.06^b^	0.18^a^	0.020	0.050
SOD, U/mL	15.00^a^	9.09^c^	12.4^ab^	0.904	0.014
GSH, μmol/mL	11.10	4.31	8.49	1.416	0.127
GSH-Px, μmol/mL	33.64	31.10	34.17	0.768	0.248

CON = piglets fed the basal diet with intraperitoneal saline injection; LPS = piglets fed the basal diet and injected intraperitoneally with lipopolysaccharide (LPS) to induce stress; EO + OA + LPS = piglets fed the basal diet supplemented with essential oils (EO) and organic acids (OA) diet and injected intraperitoneally with LPS to induce stress; TNF-α = tumor necrosis factor-α; IL = interleukin; MDA = malondialdehyde; T-AOC = total antioxidant capacity; SOD = superoxide dismutase; GSH = reduced glutathione; GSH-Px = peroxisomal glutathione.

^a-c^ Means with different letter superscripts indicate significant differences (*P*＜0.05), while with no letter or the same letter superscripts indicate no significant differences (*P*＞0.05).

### 3.3 Intestinal morphology and integrity

**Fig. 2:**
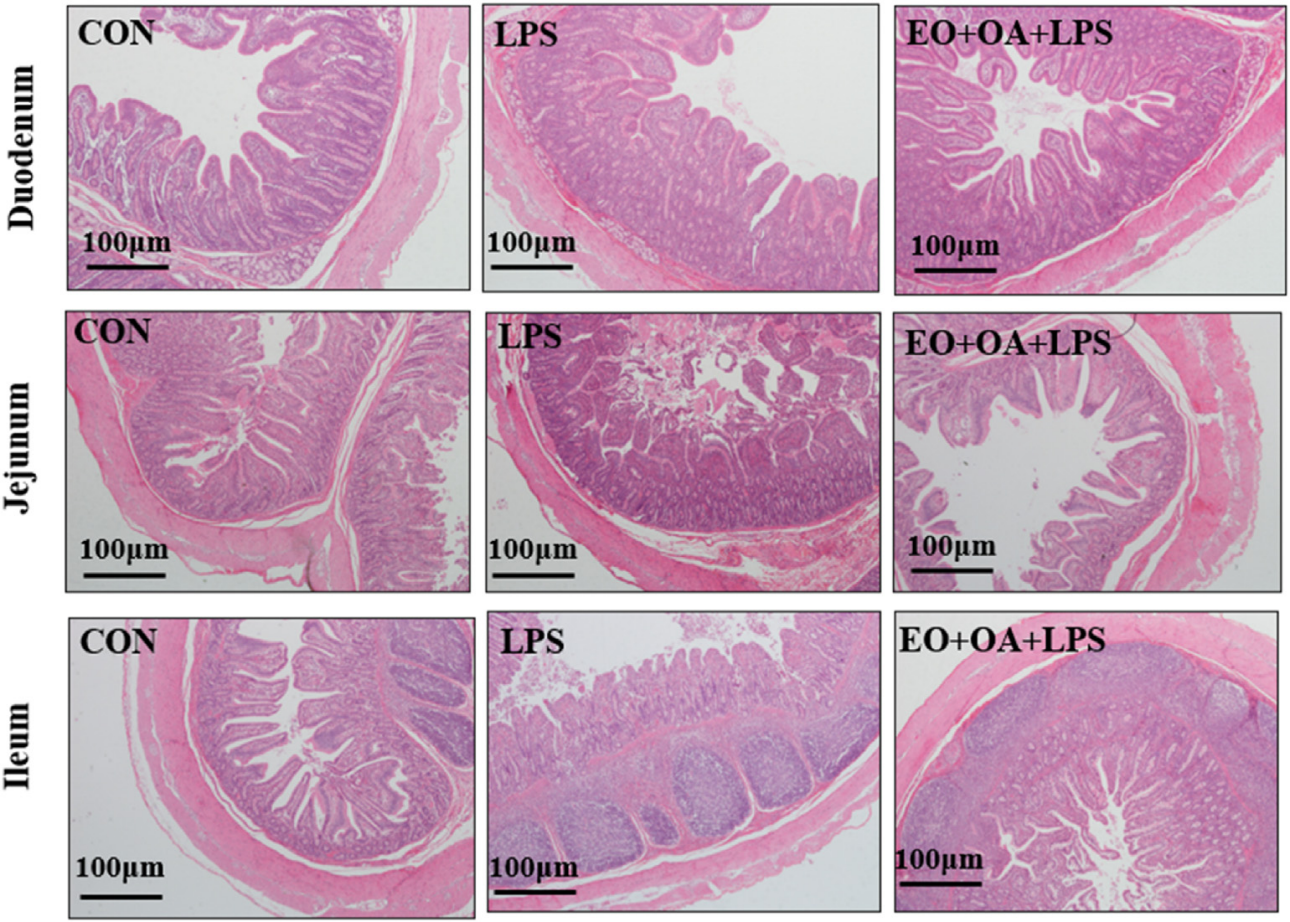
Effects of EO + OA on the intestinal morphology of weaned piglets. Representative images and histomorphometry analysis of duodenum, jejunum, and ileum tissue sections of weaned piglets stained with hematoxylin and eosin (H&E) (*n* = 6 replicates per group). CON = piglets fed the basal diet with intraperitoneal saline injection; LPS = piglets fed the basal diet and injected intraperitoneally with lipopolysaccharide (LPS) to induce stress; EO + OA + LPS = piglets fed the basal diet supplemented with essential oils (EO) and organic acids (OA) and injected intraperitoneally with LPS to induce stress.

The small intestine plays a crucial role in digestion and nutrient absorption, and its structural integrity is vital for the proper absorption of nutrients. Both the LPS group and EO + OA + LPS group exhibited varying degrees of impaired intestinal villus morphology compared to the CON group (Fig. 2). Specifically, compared with CON group, there was a significant decrease in VH and the ratio of VH to CD (VH/CD) in duodenum, jejunum and ileum segments of the small intestine (*P* < 0.05), except for VH in ileum, as well as a significant increase in CD in the LPS group (*P* < 0.001) (Table 7). Significantly higher VH and VH/CD in the duodenum and jejunum were observed in the EO + OA + LPS group compared to the LPS group (*P* < 0.05). Impaired intestinal morphology often affects the integrity of the intestinal barrier.

**Table 7:** Effects of EO + OA on the intestinal morphology of weaned piglets.

Item	CON	LPS	EO + OA + LPS	SEM	*P-*value
Duodenum					
VH, μm	453.8694^a^	378.4167^b^	446.7548^a^	7.38579	＜0.001
CD, μm	376.8281^b^	476.1345^a^	347.4435^b^	10.74564	＜0.001
VH/CD	1.2454^a^	0.8690^b^	1.3545^a^	0.38092	＜0.001
Jejunum					
VH, μm	423.1931^a^	340.3347^b^	396.2145^a^	6.75720	＜0.001
CD, μm	211.4840^b^	249.4174^a^	237.5286^a^	4.77485	＜0.001
VH/CD	2.1107^a^	1.5220^b^	1.7692^c^	0.05125	＜0.001
Ileum					
VH, μm	332.7674	303.1645	317.5302	6.76838	0.218
CD, μm	159.1087^b^	230.2422^a^	211.9188^a^	5.44862	＜0.001
VH/CD	2.2035^a^	1.5007^b^	1.6942^b^	0.05729	＜0.001

CON = piglets fed the basal diet with intraperitoneal saline injection; LPS = piglets fed the basal diet and injected intraperitoneally with lipopolysaccharide (LPS) to induce stress; EO + OA + LPS = piglets fed the basal diet supplemented with essential oils (EO) and organic acids (OA) and injected intraperitoneally with LPS to induce stress; VH = villi height; CD = crypt depth.

^a-c^ Means with different letter superscripts indicate significant differences (P＜0.05), while with no letter or the same letter superscripts indicate no significant differences (P＞0.05).

**Fig. 3:**
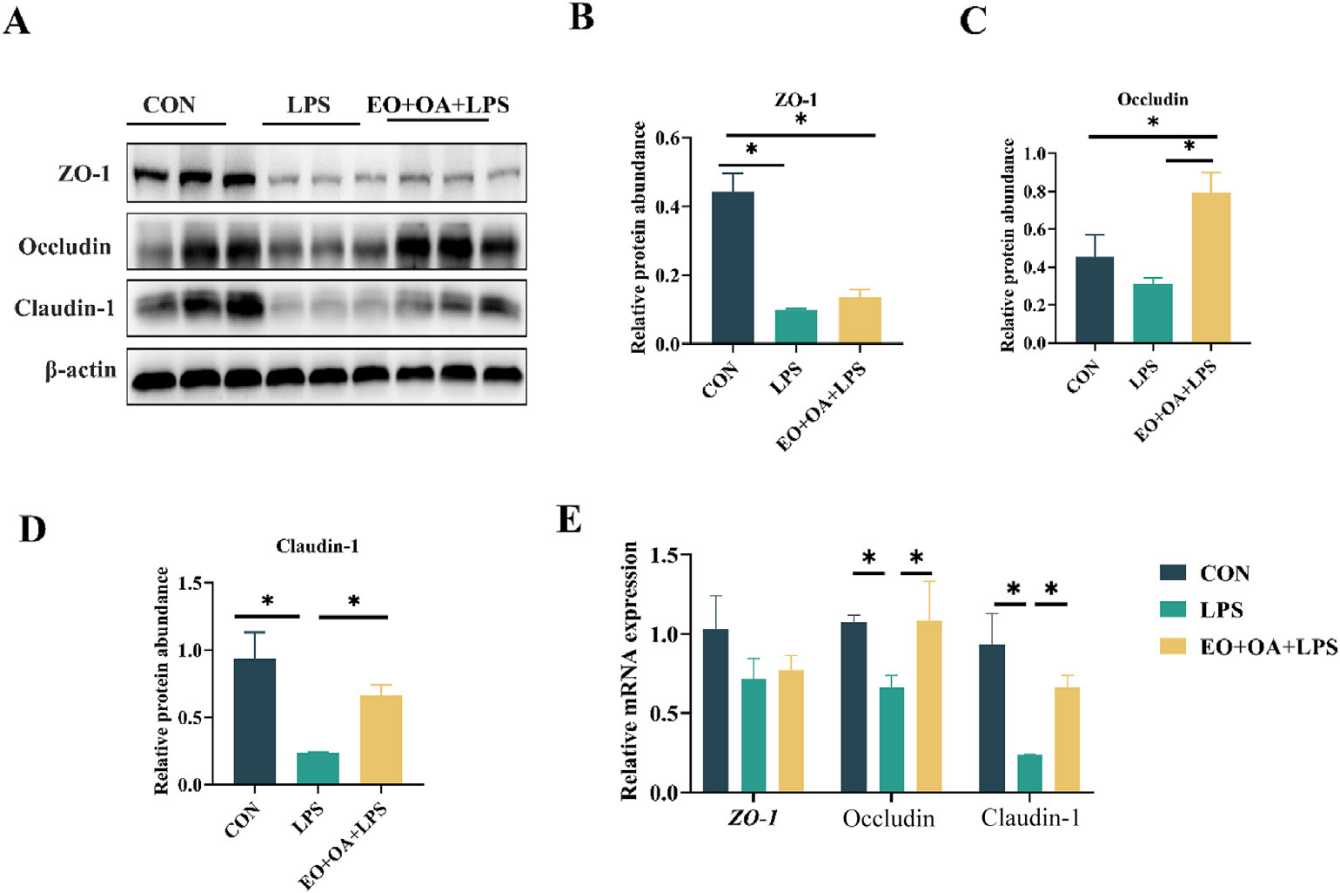
Effects of EO + OA on the intestinal integrity of weaned piglets. (A-D) Protein levels of ZO-1, occludin and claudin-1 in the jejunum (*n* = 3 replicates per group); (E) Relative mRNA expression levels of *ZO*-1, ccludino and laudinc*-*1 in the jejunum (*n* = 6 replicates per group). CON = piglets fed the basal diet with intraperitoneal saline injection; LPS = piglets fed the basal diet and injected intraperitoneally with lipopolysaccharide (LPS) to induce stress; EO + OA + LPS = piglets fed the basal diet supplemented with essential oils (EO) and organic acids (OA) and injected intraperitoneally with LPS to induce stress; ZO-1 = zonula occludens-1. ∗ means significant difference (*P* < 0.05).

To assess intestinal barrier integrity, we analyzed the expression of tight junction proteins (TJP) such as ZO-1, occludin, and claudin-1 in the jejunum. The protein levels of ZO-1 and claudin-1 were significantly reduced in the jejunum of LPS-challenged piglets compared to the CON group (*P* < 0.05) (Fig. 3B and D). However, in comparison to the LPS group, the EO + OA + LPS group exhibited significant increases in the protein levels of occludin and claudin-1 (*P* < 0.05) (Fig. 3C and D). In line with the protein expression results, the pre-supplementation of EO + OA mitigated the LPS challenge-induced reduction in ccludino and laudinc-1 mRNA relative levels (*P* < 0.05) (Fig. 3E). The results indicate that LPS challenge disrupts the normal morphology and integrity of the intestine, while supplementing with EO + OA helps maintain the normal morphology and integrity of the intestinal barrier.

### 3.4 Intestinal development

**Fig. 4:**
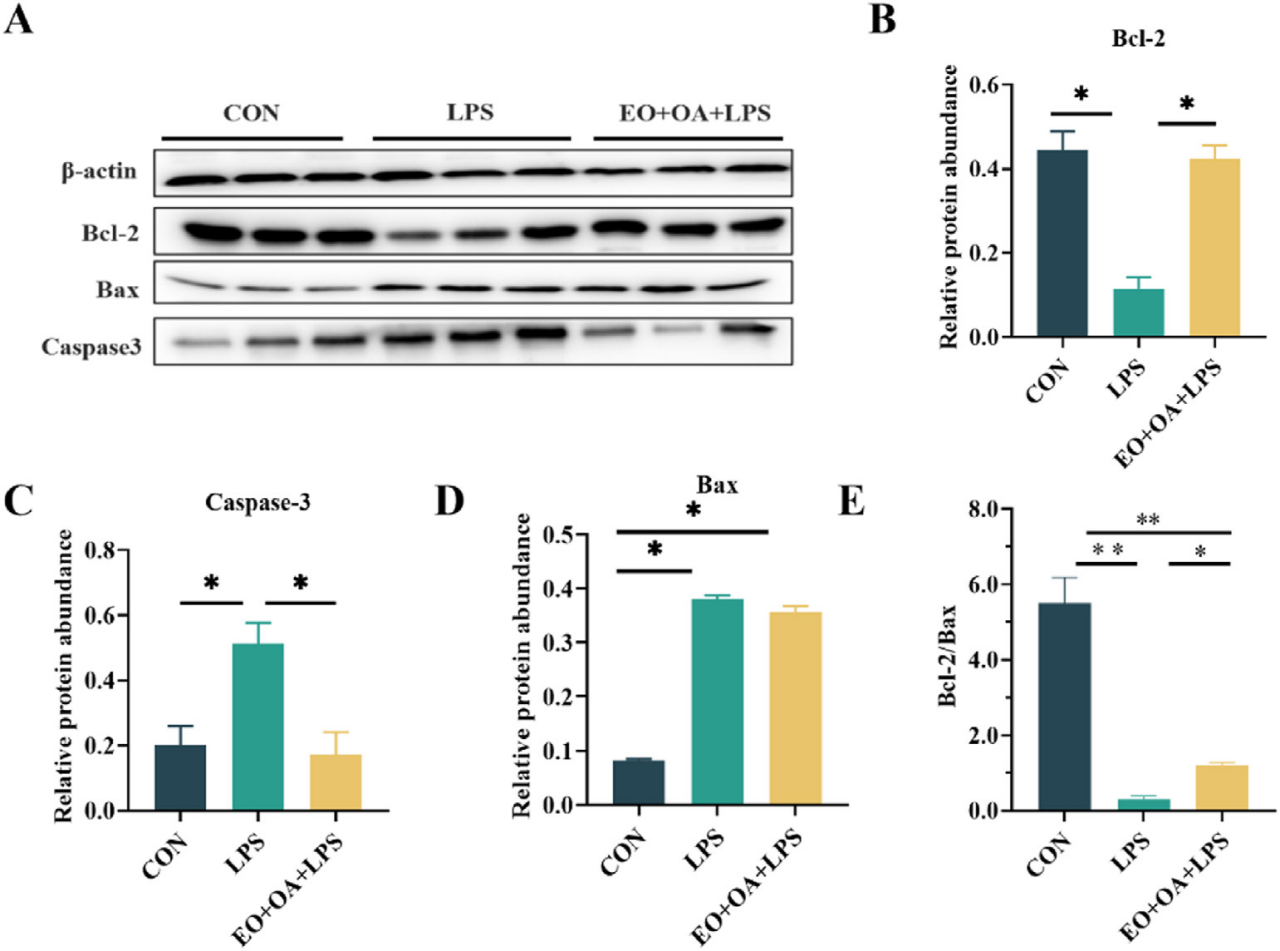
Effects of EO + OA on intestinal development of weaned piglets. (A-D) The Western blot, Bcl-2, caspase-3, and Bax relative protein expression levels (*n* = 3 replicates per group); (E) The ratio of Bcl-2 to Bax (*n* = 3 replicates per group). CON = piglets fed the basal diet with intraperitoneal saline injection; LPS = piglets fed the basal diet and injected intraperitoneally with lipopolysaccharide (LPS) to induce stress; EO + OA + LPS = piglets fed the basal diet supplemented with essential oils (EO) and organic acids (OA) and injected intraperitoneally with LPS to induce stress. Bcl-2 = B-cell lymphoma 2; caspase-3 = cysteine-aspartic proteases-3; Bax = Bcl-2-associated X protein. ∗ means significant difference (*P* < 0.05), ∗∗ means extremely significant difference (*P* < 0.01).

Compared with the CON group, the relative protein expression levels of Bax and caspase-3 were significantly higher (*P* < 0.05) in the LPS group (Fig. 4C and D), whereas the relative protein expression levels of Bcl-2 (*P* < 0.05) and Bcl-2/Bax (*P* < 0.01) was significantly lower (Fig. 4B and E). Conversely, the EO + OA + LPS group exhibited significantly reduced relative protein expression level of caspase-3 and increased relative protein expression levels of Bcl-2 and Bcl-2/Bax compared to the LPS group (*P* < 0.05) (Fig. 4B–E). The results suggest that adding EO + OA to the diet can alleviate LPS-induced intestinal developmental abnormalities, further demonstrating that pre-supplementation in the diet can improve intestinal structural development.

### 3.5 Intestinal permeability

To further confirm the ability of EO + OA to improve intestinal barrier integrity, we measured the levels of DAO, DLA, and LPS in the plasma. The results of piglets' intestinal permeability are shown in Table 8. The results demonstrated that compared to the CON group, the LPS group exhibited significantly elevated levels of LPS content, DAO activity and D-LA content (*P* < 0.05). However, compared to the LPS group, DAO activity and LPS content were significantly lower in the EO + OA + LPS group (*P* < 0.05). This further demonstrates that pre-supplementation with EO + OA can improve the intestinal barrier, preventing increased intestinal permeability that leads to elevated circulating LPS levels.

**Table 8:** Effects of EO + OA on intestinal permeability in weaned piglets.

Item	CON	LPS	EO + OA + LPS	SEM	*P-*value
LPS, EU/mL	723.1164^b^	861.4474^a^	785.9489^b^	20.39013	0.004
DAO, ng/mL	208.0990^b^	226.9153^a^	213.8539^b^	2.80119	0.003
D-LA, μmol/L	1880.1615^b^	2039.6175^a^	1944.1385^ab^	26.00665	0.021

CON = piglets fed the basal diet with intraperitoneal saline injection; LPS = piglets fed the basal diet and injected intraperitoneally with lipopolysaccharide (LPS) to induce stress; EO + OA + LPS = piglets fed the basal diet supplemented with essential oils (EO) and organic acids (OA) and injected intraperitoneally with LPS to induce stress; LPS = lipopolysaccharide; DAO = diamine oxidase; D-LA = D-lactic acid.

^a^^,b^ Means with different letter superscripts indicate significant differences (*P*＜0.05), while with no letter or the same letter superscripts indicate no significant differences (*P*＞0.05).

### 3.6 Intestinal inflammation

**Fig. 5:**
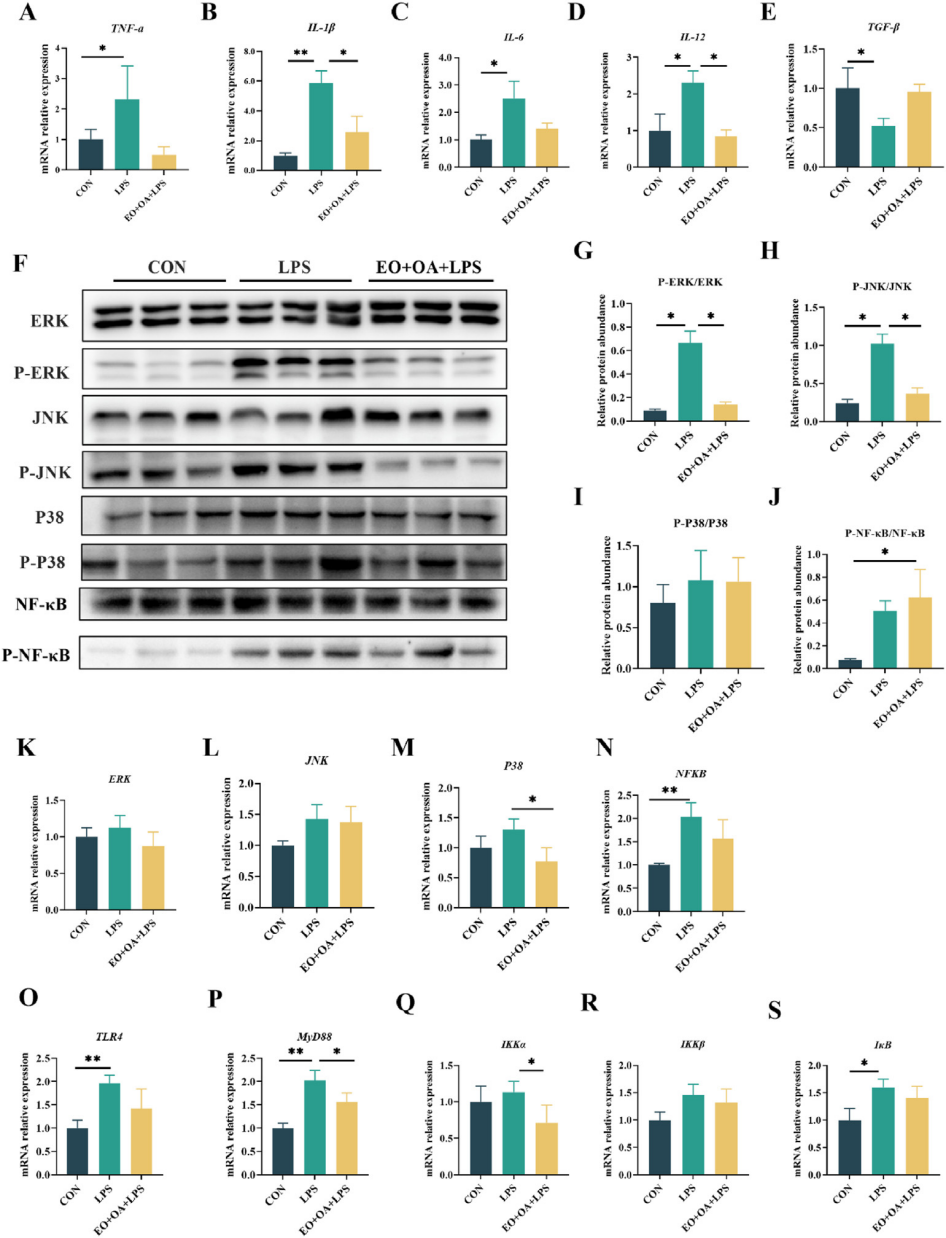
Effects of EO + OA on intestinal inflammation in weaned piglets. (A-E) Changes in intestinal inflammatory factors; (F-J) Western blotting was used to detect the protein levels of phosphorylated ERK (P-ERK), phosphorylated JNK (P-JNK), phosphorylated P38 (P-P38) and phosphorylated NF-ΚB (P-NF-ΚB) in the intestine (*n* = 3 replicates per group); (K-S) is a comparison of the mRNA relative levels of *ERK*, *JNK*, *P38*, *NF-κB*, *TLR4*, *MyD88*, *IKKα*, *IKKβ* and *IκB* (*n* = 6 replicates per group). CON = piglets fed the basal diet with intraperitoneal saline injection; LPS = piglets fed the basal diet and injected intraperitoneally with lipopolysaccharide (LPS) to induce stress; EO + OA + LPS = piglets fed the basal diet supplemented with essential oils (EO) and organic acids (OA) and injected intraperitoneally with LPS to induce stress; JNK = c-Jun N-terminal kinase; ERK = extracellular regulated protein kinases; P38 = p38 mitogen-activated protein kinase; IL-1β = interleukin-1 beta; IL-6 = interleukin-6; IL-12 = interleukin-12; TNF-α = tumor necrosis factor-α; TLR4 = Toll-like receptor 4; NF-*κ*B = nuclear factor-kappa B; MyD88 = myeloid differentiation primary response gene 88; IKK = inhibitor of kappa B kinase; IκB = inhibitor of NF-κB. ∗ means significant difference (*P* < 0.05); ∗∗ means extremely significant difference (*P* < 0.01).

Cytokines play a crucial role in influencing the integrity of the intestinal barrier. As depicted in Fig. 5, the mRNA relative expression levels of pro-inflammatory cytokines, including *TNF-α*, *IL-1β*, *IL-6*, and *IL-12*, were significantly higher in the LPS group (Fig. 5A–D) (*P* < 0.05 or *P* < 0.01), while the mRNA relative expression levels of the anti-inflammatory factor *TGF-β* were markedly lower compared to those in the CON group (Fig. 5E) (*P* < 0.05). However, in the EO + OA + LPS group, supplementation with EO and OA led to a significant reduction in mRNA relative expression levels of *IL-1β* and *IL-12* compared to the LPS group (Fig. 5B and D) (*P* < 0.05). In addition, phosphorylated JNK and ERK protein expression levels in the jejunum of piglets in the LPS group were significantly higher than those in the CON group (Fig. 5G and H) (*P* < 0.05). Conversely, the EO + OA + LPS group exhibited significantly reduced expression levels of phosphorylated ERK and JNK proteins in the jejunum compared to the LPS group (Fig. 5G and H) (*P* < 0.05). These results suggest that EO + OA supplementation has an inhibitory effect on the activation of the nuclear factor-kappa B (NF-κB) and mitogen-activated protein kinase (MAPK) signaling pathway induced by LPS. Similarly, compared to LPS piglets, the EO + OA + LPS group piglets exhibited significant downregulation in the mRNA relative expression levels of *P38*, *MyD8*8, and *IKKα* (Fig. 5M, P, and Q) (*P* < 0.05).

### 3.7 Intestinal oxidative stress

The levels of oxidative stress in the piglets' intestines are shown in Table 9. Compared to the CON group, the LPS group exhibited significantly decreased activity of SOD (*P* = 0.025), while MDA content was significantly increased (*P* = 0.006). In contrast, the EO + OA + LPS group showed significant increases in T-AOC, SOD, and GSH-Px activities, alongside a significant decrease in MDA content as compared to the LPS group (*P* < 0.05).

**Table 9:** Effects of EO + OA on intestinal oxidative stress in weaned piglets.

Item	CON	LPS	EO + OA + LPS	SEM	*P-*value
T-AOC, mmol/L	0.1206^ab^	0.0578^bc^	0.1353^a^	0.01620	0.044
MDA, nmol/mL	0.6663^b^	2.3856^a^	1.2345^b^	0.25328	0.006
SOD, U/mL	4.2189^a^	2.7980^b^	4.7485^a^	0.32472	0.025
GSH, μmol/mL	16.5004	6.0510	12.3514	1.90282	0.066
GSH-Px, μmol/mL	9.1788^b^	8.4687^b^	18.5553^a^	1.33517	＜0.001

CON = piglets fed the basal diet with intraperitoneal saline injection; LPS = piglets fed the basal diet and injected intraperitoneally with lipopolysaccharide (LPS) to induce stress; EO + OA + LPS = piglets fed the basal diet supplemented with essential oils (EO) and organic acids (OA) and injected intraperitoneally with LPS to induce stress; T-AOC = total antioxidant capacity; MDA = malondialdehyde; SOD = superoxide dismutase; GSH = reduced glutathione; GSH-Px = peroxisomal glutathione.

^a-c^ Means with different letter superscripts indicate significant differences (*P*＜0.05), while with no letter or the same letter superscripts indicate no significant differences (*P*＞0.05).

### 3.8 Intestinal microbiota composition

**Fig. 6:**
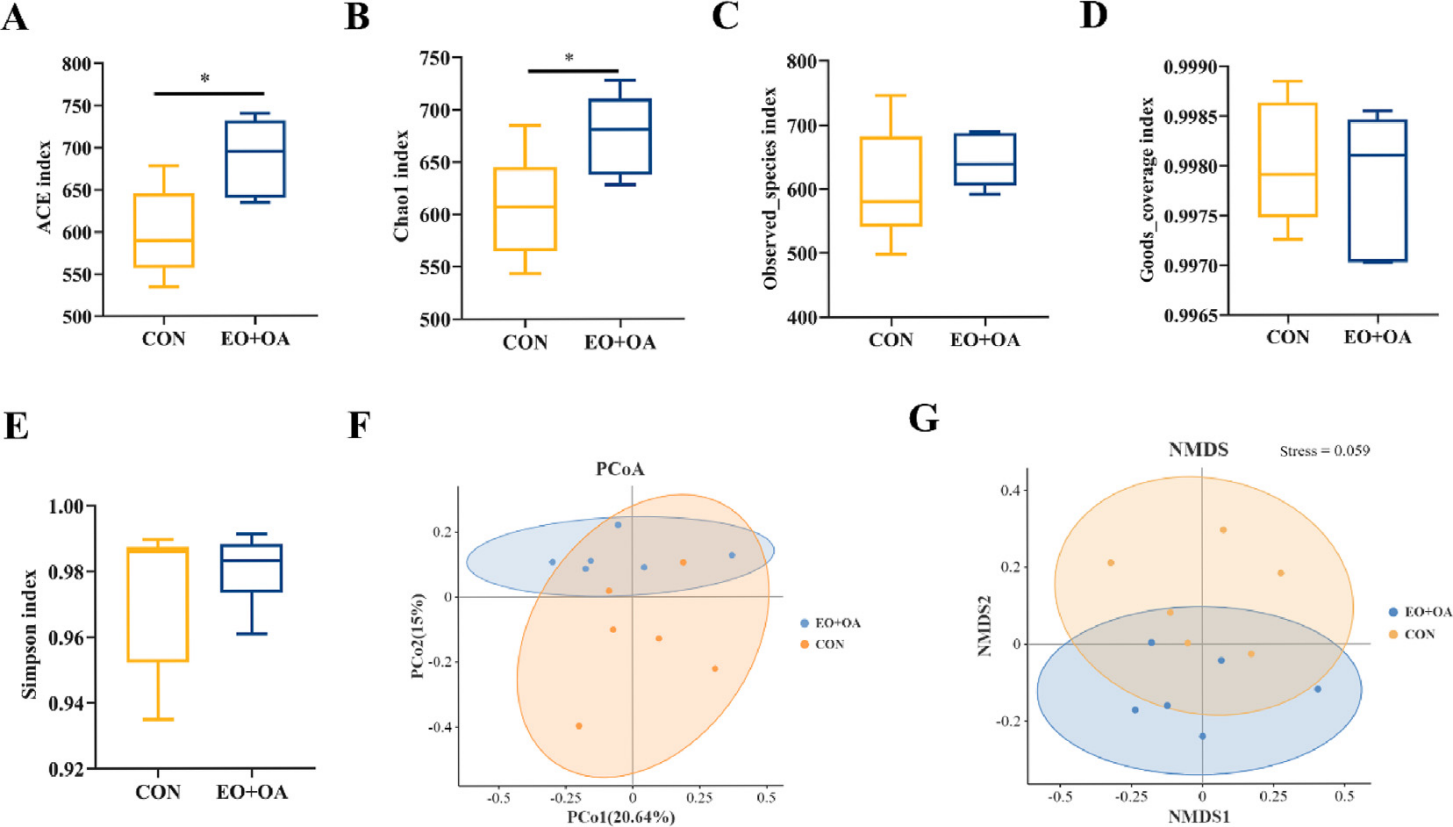
Effects of EO + OA on the diversity of gut microbiota in weaned piglets. (A-E) are alpha-diversity analyses for ACE index, Chao1 index, observed_species index, goods_coverage index, and Simpson index (*n* = 6 replicates per group); (F) Beta diversity was assessed by principal co-ordinates analysis (PCoA) at Bray–Curtis distances (*n* = 6 replicates per group); (G) Beta diversity was assessed by non-metric multidimensional scaling (NMDS) at Bray–Curtis distances (*n* = 6 replicates per group). CON = piglets fed the basic diet; EO + OA = piglets fed the basal diet supplemented with essential oils (EO) and organic acids (OA). ∗ means significant difference (*P* < 0.05).

To evaluate the impact of EO + OA on intestinal microflora, we employed 16S rRNA gene sequencing for a comparative analysis of changes in the intestinal flora of weaned piglets between the CON and EO + OA groups. Initially, to assess microbial community diversity within these groups, we measured parameters, including ACE index, Chao1 index, observed_species index, goods_coverage index, and Simpson index to evaluate α-diversity (Fig. 6A–E). Our results showed that the alpha diversity index (ACE index, Chao1 index) of microorganisms in the EO + OA group was significantly higher (*P* < 0.05) (Fig. 6A and B), while β-diversity showed no statistical difference (Fig. 6F and G).

**Fig. 7:**
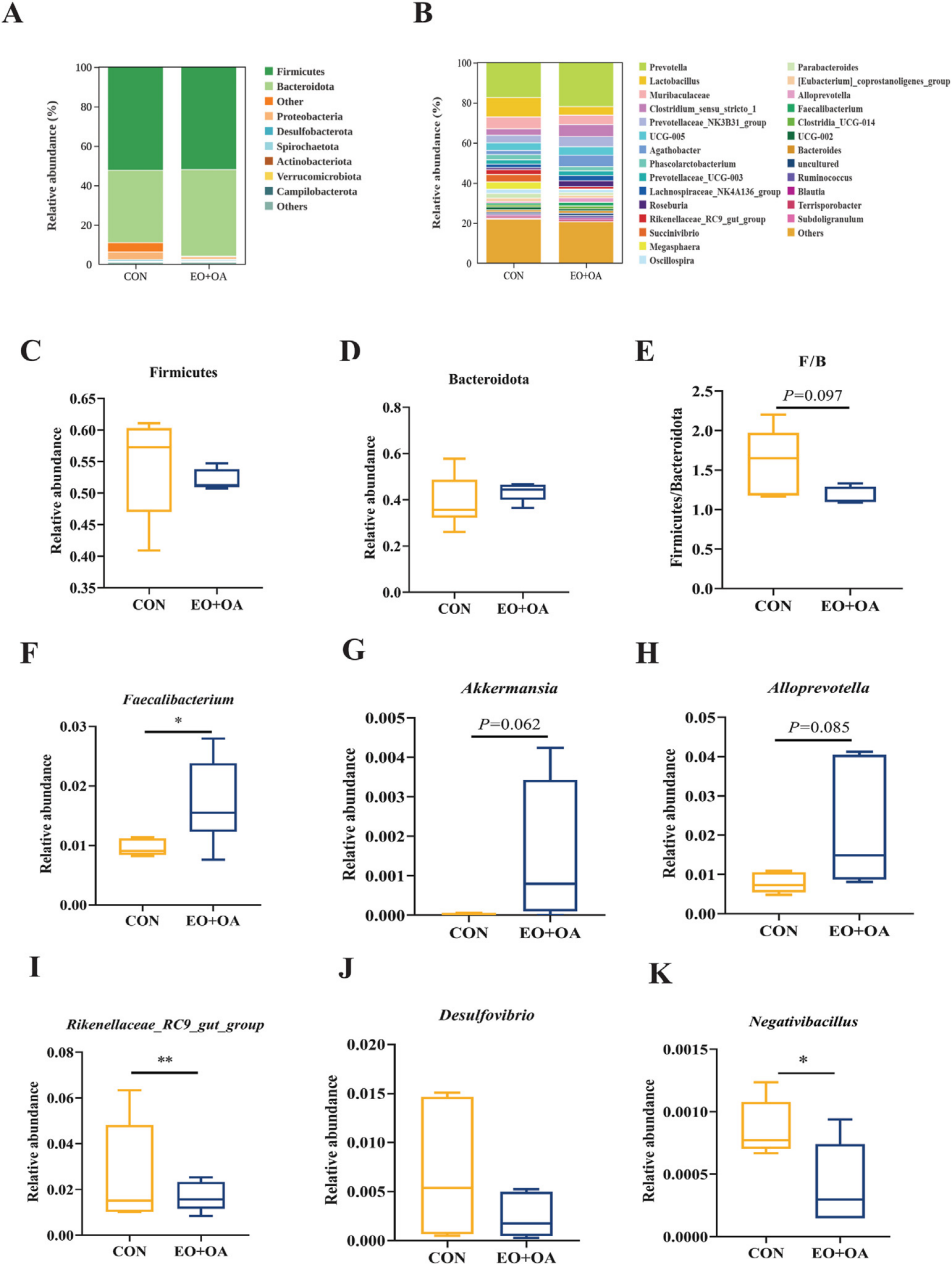
Effects of EO + OA on the diversity of gut microbiota in weaned piglets. (A) Relative abundance of microbiota at phylum level (*n* = 6 replicates per group); (B) Relative abundance of microbiota at genus level (*n* = 6 replicates per group); (C) Relative abundance of Firmicutes (*n* = 6 replicates per group); (D) Relative abundance of Bacteroidetes (*n* = 6 replicates per group); (E) Firmicutes/Bacteroidetes (F/B) (*n* = 6 replicates per group); (F-K) *Faecalibacterium*, *Akkermansia*, *Alloprevotella*, *Rikenellaceae_RC9_gut_ group, Desulfovibrio* and *Negativibacillus*, between the CON group and the EO + OA group abundance (*n* = 6 replicates per group). CON = piglets fed the basic diet; EO + OA = piglets fed the basal diet supplemented with essential oils (EO) and organic acids (OA). ∗ means significant difference (*P* < 0.05); ∗∗ means extremely significant difference (*P* < 0.01).

Subsequently, to explore alterations in microbial structure between the two groups, we conducted a comparative analysis of the overall microbial composition at both the phylum and genus levels (Fig. 7A and B). The relative abundance of Bacteroidotas and Firmicutes, as well as the F/B ratio, changed in the EO + OA group compared to the CON group (Fig. 7C–E). Furthermore, microbial analysis indicates an increase in relative abundance of *Faecalibacterium* (*P* < 0.05) in the EO + OA group compared to the CON group, with a rising trend observed in relative abundance of *Akkermansia* (*P* = 0.062) and *Alloprevotella* (*P* = 0.085) (Fig. 7F–H). In contrast, harmful bacteria in the EO + OA group, specifically the relative abundance of *Rikenellaceae_RC9_gut_group* (*P* < 0.01) and *Negativibacillus* (*P* < 0.05), exhibited a significant decrease (Fig. 7I and K). The relative abundance of *Desulfovibrio* showed no statistically significant difference between the two groups (*P* > 0.05). Our findings reveal the beneficial effects of EO + OA on increasing gut microbial diversity and improving specific microbial taxa in the gut microbiota.

### 3.9 Correlation analysis

**Fig. 8:**
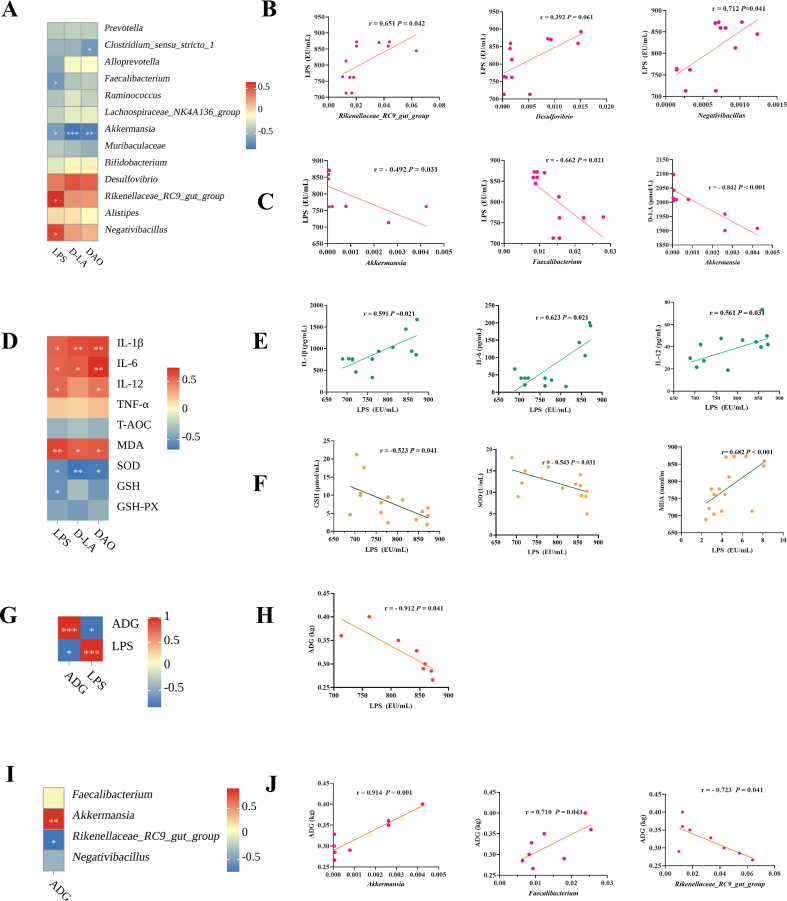
Correlation analysis between lipopolysaccharide (LPS) content and intestinal leakage indicators, intestinal inflammatory factors, and correlation analysis between plasma LPS content and intestinal microbiota. (A) Correlation of gut microbes with plasma LPS content and the gut barrier. (B) Linear correlation analysis with plasma LPS content for intestinal bacteria (*Rikenellaceae_RC9_gut_gorup*, *Desulfovibrio, Negativibacillus*). (C) Linear correlation analysis with plasma LPS content for *Faecalibacterium and Akkermansia,* and linear correlation analysis with plasma D-LA content for intestinal bacteria *Akkermansia*, respectively. (D) Correlation analysis of plasma LPS content with plasma oxidative stress and plasma inflammation in the body. (E) Linear correlation analysis with plasma LPS content for plasma IL-β， IL-6 and IL-12 contents, respectively, in turn. (F) Linear correlation analysis with plasma LPS content for plasma MDA content, SOD activity, and GSH content, respectively, in turn. (G-H) Analysis of the correlation between plasma LPS content and piglet ADG. (I-J) Analysis of the correlation between intestinal microorganisms and the ADG of piglets. LPS = lipopolysaccharide; DAO = diamine oxidase; D-LA = D-lactic acid; TNF-α = tumor necrosis factor-alpha; IL-1β = interleukin-1 beta; IL-6 = interleukin-6; IL-12 = interleukin-12; MDA = malondialdehyde; SOD = superoxide dismutase; GSH = reduced glutathione; GSH-Px = peroxisomal glutathione; ADG = average daily gain. ∗ means significant correlation (*P*＜0.05), ∗∗ means very significant correlation (*P*＜0.01), ∗∗∗ means extremely significant correlation (*P*＜0.001).

To investigate whether the improvement of the intestinal barrier by the mixture of EO and OA is associated with changes in the gut microbiota, we conducted a correlation analysis among LPS, gut microbes, gut barrier, organismal oxidative stress, and inflammation. The heat map and linear correlation scatter plot revealed a positive correlation between LPS and the relative abundance of *Rikenellaceae_RC9_gut_group* (r = 0.651, *P* = 0042), *Negativibacillus* (r = 0.712, *P* = 0.041), and *Desulfovibrio* (r = 0.392, *P* = 0.061), while showing a negative correlation with the relative abundance of *Akkermansia* (r = −0.492, *P* = 0.031) and *Faecalibacterium* (r = −0.662, *P* = 0.021) (Fig. 8A–C). We also observed a significant negative correlation between the relative abundance of *Akkermansia* and intestinal permeability (D-LA) (r = −0.842, *P* ＜ 0.001) (Fig. 8A and C).

The heat map showed a significant positive correlation between LPS and inflammatory factors and oxidative stress in plasma (Fig. 8D). The plasma LPS content was linearly and positively correlated with MDA (r = 0.682, *P* ＜ 0.001) content, and negatively correlated with SOD (r = −0.543, *P* = 0.031) activity and GSH (r = −0.523, *P* = 0.041) content (Fig. 8F). LPS is positively correlated with plasma inflammatory factors IL-6 (r = 0.623, *P* = 0.021), IL-1β (r = 0.591, *P* = 0.021) and IL-12 (r = 0.561, *P* = 0.031) contents (Fig. 8E), and negatively correlated with weaned piglets ADG (r = −0.912, *P* = 0.041) (Fig. 8G and H). Additionally, we conducted analyses correlating microbial composition with ADG in weaned piglets and observed a positive correlation between ADG and the relative abundance of *Akkermansia* (r = 0.914, *P* = 0.001), as well as *Faecalibacterium* (r = 0.710, *P* = 0.043) (Fig. 8I). The relative abundance of *Rikenellaceae_RC9_gut_group* (r = −0.723, *P* = 0.041) was negatively correlated with ADG (Fig. 8J).

## 4 Discussion

Stressful weaning usually leads to intestinal dysfunction, gut microbial imbalance, inflammation (Tang et al., 2022), and oxidative stress, ultimately resulting in reduced piglet growth performance (Moeser et al., 2017; Smith et al., 2010). Essential oils and OA are both effective additives for alleviating this damage during the weaning process without causing resistance or environmental contamination (Li et al., 2018; Ma et al., 2022; Vardar-Unlu et al., 2003). The combined use of these two distinct functional substrates has shown synergistic benefits for the intestinal health and performance of young animals due to the different mechanisms of their action (Cui et al., 2024; Ma et al., 2022; Wang et al., 2024). The present study revealed that dietary supplementation with EO and OA can enhance the ADG in weaned piglets while reducing the F/G. This finding is consistent with results observed in other animal species, further demonstrating the advantageous effects of these two additives and suggesting a highly efficient method of their application in the pig industry.

Antioxidative imbalance and elevated inflammation are often observed in piglets suffering from weaning stress, and these disorders are both inducers of higher vulnerability and susceptibility to pathogens (Dang et al., 2022; Lauridsen, 2019). Studies have shown that supplementing the diet of weaned piglets with antioxidants such as polyphenols, oregano essential oil, and vitamin E could enhance overall and intestinal redox balance and improve inflammation status, alleviating the adverse effects of weaning and leading to improved growth performance (Frame et al., 2020; Hao et al., 2021). Here, we observed a decrease in plasma antioxidant enzyme levels and an increase in inflammatory markers (TNF-α, IL-1β, and IL-6) in the LPS group, while this disturbance was greatly alleviated by the supplementation of EO and OA. Besides, the supplementation of EO and OA also ammoniate the LPS-induced elevation of intestinal oxidative stress and inflammation, which consistent with the findings in plasma, suggesting their benefits in alleviating stress response induced by LPS. Toll-like receptor 4 is a critical receptor that mediates inflammation by detecting and binding to LPS (Zhang et al., 2022), subsequently activating the MAPK and NF-κB signaling pathways (Lv et al., 2021). This activation initiates intracellular inflammatory and immune responses, resulting in the production of inflammatory factors and exacerbating organismal inflammation (Stephens and von der Weid, 2020). Previous experiments have observed that intraperitoneal injection of LPS activates the TLR4/NF-κB/MAPK signaling pathway in mice (Li et al., 2023), triggering an inflammatory response. In addition, in another study, dietary addition of EO + OA was observed to inhibit the activation of intestinal NF-κB signaling pathway in weaned piglets (Yang et al., 2020a). Consistently, LPS activated the TLR4/NF-κB/MAPK signaling pathway, and the addition of EO + OA reduced the activation to control levels. These results suggest that EO + OA's alleviation of stress in piglets may be achieved by inhibiting the activation of the TLR4/NF-κB/MAPK signaling pathway.

The lining of the intestinal tract consists of a single layer of epithelial cells that form a physical barrier to prevent harmful substances from entering the intestinal tissue (He et al., 2019). Acute inflammation has been widely reported to disrupt the integrity and barrier function of the intestinal structure (Guo et al., 2022; Li et al., 2023) and increase intestinal permeability, leading to the development of leaky intestines (Zhao et al., 2021). In the current study, supplementation of EO + OA effectively alleviated extensive intestinal damage induced by LPS exposure in weaned piglets, as indicated by improvements in intestinal morphometric indices (VH and VH/CD) in the duodenum, jejunum, and ileum, alongside a reduction in apoptosis markers (Bax and Caspase-3). Plasma D-LA content and DAO activity can serve as biomarkers of intestinal barrier integrity (Cui et al., 2021). Lipopolysaccharide, an endotoxin located on the surface of Gram-negative bacterial cell walls in the intestine, can trigger a systemic inflammatory response (Stephens and von der Weid, 2020; Yang et al., 2021), with an upregulation of its levels in plasma potentially indicating heightened intestinal permeability indirectly (Nesci et al., 2023; Wang et al., 2023). Our research findings indicated that the elevated plasma levels of D-LA, DAO, and LPS in LPS-challenged piglets approached levels similar to those of the control group, indicating a restoration of intestinal permeability with the addition of EO and OA mixture. Tight junction proteins (ZO-1、Occludin and Claudin-1) play a crucial role in regulating intestinal permeability and maintaining the structure and integrity of the intestinal barrier (Hu et al., 2020). A decrease in these proteins indicates a disruption of the intestinal barrier (He et al., 2019). In our study, we observed a decrease in TJP expression in piglets subjected to LPS challenge, yet the combined application of EO and OA effectively alleviated the decline in jejunal TJP, suggesting restored intestinal integrity. The above findings suggest that EO + OA effectively mitigated LPS-induced impairments in intestinal development and barrier integrity in weaned piglets, thereby reducing intestinal permeability and preventing the leakage of external toxic substances into the circulation.

The intestinal microbiota plays a crucial role in the physiological and health status of the host (Ding et al., 2022) and serves as a biological barrier protecting against pathogens (Hills et al., 2019; Nagpal et al., 2016). Dysbiosis of the gut microbiota, especially an increase in harmful bacteria, leads to the production of more toxic metabolites, such as hydrogen sulfide and LPS, with the potential for translocation into the systemic circulation (Gabler et al., 2018; Hu et al., 2020). Organic acids are deemed effective for regulating gut health, especially when combined with EO, which modify bacterial cell structure (Satterlee et al., 2023), enabling OA to enter the bacterial cell membrane and damage specific harmful bacteria (Yang et al., 2020). Here, we observed that at the genus level, the addition of EO + OA altered the specific intestinal microbial abundances in weaned piglets. The relative abundances of *Negativibacillus*, *Desulfovibrio*, and *Rikenellaceae*_*RC9_gut_group* decreased, while *Faecalibacterium*, *Akkermansia*, and *Alloprevotella* showed a significant increase. *Rikenellaceae_RC9_gut_group*, *Desulfovibrio*, and *Negativibacillus* are all gram-negative bacilli recognized for their notable capacity to produce LPS (An et al., 2023; Emami et al., 2021; Wang et al., 2022). They have been reported to exhibit a higher abundance in the intestines of patients with inflammatory bowel disease and are associated with elevated serum LPS contents (An et al., 2023; Wang et al., 2022). Here, the reduction of these bacteria in the EO + OA group may be one of the main reasons for the decreased plasma LPS contents, as we have previously demonstrated by reducing intestine-derived LPS production. Besides, some gut bacteria have been shown to have a positive influence on intestinal health and barrier integrity (Li et al., 2022; Nesci et al., 2023; Ottman et al., 2017). *Akkermansia* is a promising probiotic candidate due to its metabolic byproducts which can interact with and strengthen the intestinal barrier (Ottman et al., 2017). *Faecalibacterium* is known for its capacity to produce butyric acid, which serves as an energy source for intestinal cells and helps maintain the integrity of the intestinal mucosal barrier (Zhu et al., 2022). The elevation of these two bacteria in the group supplemented with EO + OA suggests a potential enhancement of intestinal barrier function. In summary, these results suggest that dietary supplementation with EO + OA may safeguard the intestinal barrier by increasing beneficial bacteria (*Faecalibacterium*, *Akkermansia*, and *Alloprevotella*) and reducing harmful LPS producing bacteria *(Rikenellaceae_RC9_gut_group*, *Negativibacillus*, *Desulfovibrio*), thereby reducing the intestine-derived LPS production and preventing its translocation through the leaky barrier into circulation.

We further performed correlation analysis between plasma LPS content and the intestinal microbiota, the intestinal barrier, as well as oxidative stress and inflammation indicators, with the purpose of exploring potential causal relationships between plasma LPS content and piglet performance. The negative correlation observed between the relative abundance of *Akkermansia* and intestinal permeability suggests its protective role in maintaining intestinal integrity. Moreover, the positive association of the relative abundance of *Rikenellaceae_RC9_gut_group* and *Negativibacillus* with plasma LPS content suggests that the elevation in plasma LPS content may be attributed to the increased abundance of these bacteria. Plasma LPS contents exhibited positive correlations with inflammatory factors and negative correlations with antioxidant capacity and piglet performance (ADG), highlighting the substantial impact of LPS on health outcomes. Additionally, the positive correlations of ADG with the relative abundance of *Akkermansia* and *Faecalibacterium*, alongside the negative correlation of the relative abundance of *Rikenellaceae_RC9_gut_group*, indicate the potential of microbiota modulation in promoting growth and development. Our correlation analysis between LPS and various factors reveals potential causal relationships and underscores the significance of gut microbiota in piglet performance.

## 5 Conclusion

In conclusion, dietary addition of EO and OA prevented the leakage of LPS into the circulation by improving intestinal barrier integrity and microbiota composition with increased beneficial bacteria and decreased LPS-producing bacteria, while also enhancing the antioxidant and anti-inflammatory abilities of weaned piglets via inhibiting the activation of the TLR4/NF-κB/MAPK signaling pathway, ultimately improving the growth performance. These findings highlight for the first time the critical negative role of intestine derived LPS in weaning stress and its importance as a potential nutritional intervention target in the future.

## Author contributions

**Fang Chen, Shihai Zhang** and **Wutai Guan:** Conceptualization, Methodology, Software. **Yibo Wang:** Writing- Original draft preparation. **Tanyi Deng** and **Wen Ma:** Visualization, Investigation. **Zhichao Fu** and **Yueqi Zhao:** Data curation. **Xuemei Zhou** and **Yulong Wei:** Software, Validation. **Xiaoyu Zheng** and **Fang Chen:** Writing- Reviewing and Editing.

## Declaration of competing interest

We declare that we have no financial and personal relationships with other people or organizations that can inappropriately influence our work, and there is no professional or other personal interest of any nature or kind in any product, service and/or company that could be construed as influencing the content of this paper.
